# Access to neighborhood destinations that offer opportunities for physical activity and socialization is associated with favorable post-stroke outcomes

**DOI:** 10.1016/j.jstrokecerebrovasdis.2026.108545

**Published:** 2026-01-02

**Authors:** Leanna M Delhey, Jon Zelner, Xu Shi, Lewis B Morgenstern, Devin L Brown, Melinda A Smith, Erin C Case, Lynda D Lisabeth

**Affiliations:** a Department of Epidemiology, University of Michigan School of Public Health, Ann Arbor, MI, USA; b Department of Biostatistics, University of Michigan School of Public Health, Ann Arbor, MI, USA; c Stroke Program, University of Michigan Medical School, Ann Arbor, MI, USA

**Keywords:** Stroke, Survivor, Cognition, Depression, Activities of Daily Living, Quality of Life, Neighborhood

## Abstract

**Objective::**

Assess associations between destinations near stroke survivor’s residence – places like restaurants, recreation centers, and stores that offer opportunities for physical activity and socialization outside of the home and work – and their poststroke outcomes.

**Methods::**

We included non-Hispanic white and Mexican American incident stroke survivors enrolled in the Brain Attack Surveillance in Corpus Christi project (2009–19), a population-based cohort in Texas. Exposure: count of destinations within 0.5-miles around survivors’ residences. Outcomes assessed at approximately 3-, 6-, and 12-months poststroke: cognition (Modified Mini-Mental State Examination), functioning (activities of daily living (ADL)/instrumental ADL), health-related quality of life (abbreviated Stroke-Specific Quality of Life scale), and depression (Patient Health Questionnaire-8). We fit adjusted linear mixed models and considered interactions with follow-up time and stroke severity (NIH stroke scale - mild (<5), moderate-severe (≥5)).

**Results::**

We included 1,786 survivors who completed 3 (*N* = 1,321), 6 (*N* = 677), or 12-month interviews (*N* = 652). Median age was 64 years, 55% male, and 74% mild stroke. Stroke severity modified associations with functioning (*p* = 0.09) and quality of life (*p* = 0.05), follow-up time did not (*p* > 0.25). Among moderate-severe stroke survivors, more destinations were associated with more favorable functioning (mean difference=−0.12, 95% CI= −0.22, −0.01) and quality of life (mean difference=0.16, 95% CI=0.03, 0.30). No associations were observed among mild stroke survivors or with cognition or depression (*p* > 0.05).

**Interpretation::**

Among moderate-severe stroke survivors, more nearby destinations were associated with more favorable functioning and quality of life in the first year. Future research is needed to explore if specific types of destinations may support more favorable outcomes.

## Introduction

Recent findings from the Behavioral Risk Factor Surveillance System indicate, in 2020–2022, the age-standardized prevalence of self-reported stroke was 2.9%, a 7.8% increase since 2011–2013.^1^ Among older adults ≥65 years, 7.7% self-reported having had a stroke in 2020–2022.^1^ Stroke is a leading cause of long-term disability.^2^ Research suggests survivors experience cognitive impairment, reduced functioning, more frequent depressive symptoms, and report lower quality of life relative to others of similar age without stroke and that this persists in the year post-stroke.^3–6^ The effectiveness of recovery efforts is hypothesized to relate to factors concerning the survivor, their caregiver(s), their medical treatment and rehabilitation program, and the community they live in.^7–9^

Because of increased limitations on functioning and mobility, a stroke survivor’s residential neighborhood can be particularly influential in their outcomesoutcomes.^10,11^ Community-dwelling stroke survivors recognize the importance of their neighborhood when discussing recovery-related unmet needs, such as lack of space for physical activity and opportunity to socialize.^9,12,13^ Qualitative research suggests residing near destinations that offer opportunities for physical activity and socialization can promote better poststroke outcomes.^14,15^ Specific neighborhood destinations have been shown to be beneficial for older adult stroke survivors along multiple dimensions including cognition, mental health, and subjective well-being.^9,14–24^ These include entertainment venues, museums & libraries, recreational facilities, personal care locations (e.g., salons or laundromats), eateries and bars, stores (e. g., grocery stores, superstores, and retail shops), religious, civic, and social organizations, post offices, banks, and social service sites like senior centers.^9,14–24^ However, there is limited quantitative research on the association between neighborhood destinations and poststroke cognition, functioning, quality of life, or depression.^23,25,26^ We previously reported greater density of destinations within the stroke survivor’s residential census tract was associated with better cognition among stroke survivors at 3 months poststroke.^23^ Additionally, both destinations overall, and more specifically recreation centers, were associated with better functioning and quality of life 3-months following stroke among survivors of a moderate-severe stroke.^23,25^ In contrast, Twardzik et al., reported that a composite measure of neighborhood destinations, land use, and street aesthetics was not predictive of quality of life at 6-months to 3-years poststroke.^26^ Previous studies conducted by Delhey et al. were limited by defining neighborhood using the residential census tract and only evaluating outcomes at 3-months post-stroke.^23,25^ The study by Twardzik et al. did not consider the influence of destinations separate from other aspects of the neighborhood environment and did not evaluate other poststroke outcomes of cognition, functioning, or depression.^26^

To address these limitations, we aimed to identify associations between the availability of neighborhood destinations within a half-mile radius of the survivor’s residence with poststroke health outcome measures of cognition, functioning, quality of life, and depression. We conducted a study using available data from a population-based cohort of stroke survivors from a bi-ethnic urban community and linked this information to the National Establishment Time Series (NETS), a commercially available dataset on establishments in the study region.

## Methods

Our study population was drawn from the Brain Attack Surveillance in Corpus Christi (BASIC) project. The BASIC project is a population-based surveillance cohort of individuals with stroke, ≥45 years of age, and residing ≥6 months in Nueces County, Texas. This is an ethnically diverse community of ~350,000 people, 61% of whom were Hispanic as of 2020.^27^ Potential stroke cases were identified through active surveillance of daily admission logs, medical and intensive care units, and passive surveillance of discharges from emergency departments and hospitals.^3,28^ All potential cases were validated by a stroke neurologist. All participants of the BASIC project or their proxy (e.g., next of kin) provided written informed consent, and the study was reviewed and approved by the institutional review boards at the University of Michigan and two local hospital systems (HUM0041536, first approved in 1999). We adhered to the Strengthening the Reporting of Observational Studies in Epidemiology Reporting Guidelines (STROBE).^29^

The BASIC project collected extensive data from medical records and interviews conducted in English or Spanish shortly following the stroke event (baseline) and approximately 3-, 6-, and 12-months poststroke (only those enrolled in 2014–19 were eligible to complete the 6- and 12-month follow-up).^5^ Baseline and follow-up interviews were completed by the patient or a proxy. At baseline, information was obtained by medical record abstraction on age, sex, insurance status, prior comorbidities, smoking, excessive alcohol use, and initial stroke type. Initial stroke severity was assessed using the NIH stroke scale (NIHSS) obtained from the medical records or by validated algorithm.^30,31^ We considered comorbidities of amyotrophic lateral sclerosis, atrial fibrillation, cancer, chronic obstructive pulmonary disease, coronary heart disease or myocardial infarction, dementia or Alzheimer’s disease, diabetes, end-stage renal disease, epilepsy, heart failure, hyperlipidemia, hypertension, and Parkinson’s disease. Additional information obtained by interview included race/ethnicity, educational attainment, marital status, a social support scale, and pre-stroke health. We included information from medical records and baseline interviews as individual- and interpersonal-level covariates. We also incorporated neighborhood-level covariates by linking data on socioeconomic status (disadvantage and affluence score) and ethnic and immigrant composition from the National Neighborhood Data Archive (NANDA).^32^ These factors were linked according to the census tract in where the survivor resided at the time of stroke and the 5-year period during which the stroke occurred.

At follow-up interviews, measures of function, cognition, depression, and health-related quality of life were obtained. Function was assessed as the average self-rated difficulty of 7 activities of daily living (ADL) and 15 instrumental ADL (IADL; range: 1 to 4; lower scores indicate poorer functioning) items..^3^ Cognitive performance was assessed using the Modified Mini-mental State Exam (3MSE) which is a measure of global cognitive function (range: 0–100; higher scores represent better cognitive performance).^3^ Depressive symptoms were assessed by self-report on the Patient Health Questionnaire Eight (PHQ8), a validated depression severity measure (range: 0–24; lower scores indicate fewer depressive symptoms).^33^ The abbreviated Stroke-Specific Quality of Life scale (SS-QoL) was used to assess physical and psychosocial dimensions of quality of life (range: 1–5; higher scores indicate more favorable health-related quality of life).^34^ The 3MSE and PHQ8 were not completed in interviews conducted with a proxy.

We included BASIC project enrollees with a first-time ischemic or hemorrhagic stroke from 2009 to 2019. We excluded individuals who were institutionalized prior to stroke, did not complete the baseline interview, or whose baseline interview was completed by a proxy, as proxies could not provide valid responses across all baseline instruments. Residential address had to be successfully geocoded. We further restricted analyses to those who self-identified as Mexican American or Non-Hispanic White due to small sample sizes in other racial/ethnic groups. Follow-up interviews were conducted at approximately 3, 6, and 12 months poststroke. Survivors enrolled in 2014–2019 were eligible for 6 and 12-month assessments. Out-come-specific exclusions were applied: the 3MSE and PHQ-8 were not assessed in proxy interviews; SS-QoL was not collected for those enrolled in 2009; PHQ8 was not collected for those enrolled in 2009–2010.

We used NETS data to identify potential neighborhood destinations during the year of the survivor’s stroke. We obtained data on neighborhood-level confounders from the American Community Survey. Our exposure was the count of neighborhood destinations within a 0.5 mile radius of the survivor’s residence as documented during their initial admission for stroke. This measure only assessed destinations the survivor might have the opportunity to frequent and did not assess actual utilization or accessibility of the destinations. Based on previous publications, we selected destination types which were associated with positive outcomes, or were highlighted in qualitative research as potentially beneficial among either stroke survivors or all older adults for similar outcomes.^9,14–25^ We identified these destinations using the North American Industry Classification System, previously described.^25^ Briefly, destination types included retail and food stores; postal offices and banks; social service sites; arts, intellectual, entertainment, and recreation centers; eating and drinking establishments; personal care providers; and religious, civic, and social organizations. We used ArcGIS to count the number of active destinations within a 0.5-mile radius of the survivor’s residence for the calendar year in which the stroke occurred.

We described the study population’s baseline characteristics and outcome measures according to the quartiles of destination count. Continuous variables were summarized by median and interquartile range (IQR); categorical variables by counts and percentages. We tested for differences by quartile of destinations in continuous characteristics with analysis of variance or categorical characteristics with chi-squared tests. We fit linear mixed models for each outcome. We included outcomes for each time point and accounted for repeated measures in the model. To account for non-completion of the baseline interview, completion of the baseline interview by proxy, and non-completion of follow-ups, we applied inverse probability weighting. We conducted multiple imputation with chained equations to account for missing data and created 10 imputed datasets for analysis. We pooled results from imputed datasets using Rubin’s rule.^35^ To aid in interpretation, we centered the count of destinations at the median and standardized by the interquartile range. The estimated difference in the outcome measure reflects the mean difference expected for the 75^th^ versus the 25^th^ percentile of destination count. Models were sequentially adjusted for 1) demographics: age quartile, sex, race/ethnicity; 2) socioeconomic status (SES): educational attainment, insurance; 4) pre-stroke health: modified Rankin scale (mRS), informant questionnaire on cognitive decline in the elderly (IQCODE), depression, comorbidity score, 5) pre-stroke behaviors: excessive alcohol use, smoking; 6) interpersonal factors: marital status, social support score; 7) neighborhood factors: neighborhood disadvantage score, neighborhood affluence score, neighborhood ethnic/immigrant score; and 8) stroke characteristics: initial stroke severity and type. We dichotomized stroke severity into mild (NIHSS<5) or moderate-severe (NIHSS≥5). We included interaction terms between stroke severity and the exposure to consider effect modification by stroke severity and/or follow-up time. We interpreted results based on effect sizes from model coefficients, and considered 95% confidence intervals (CI), and p-values for main and interaction effects (*p* < 0.05 and *p* < 0.15, respectively) as tools to understand relative uncertainty in these estimates.^36,37^ We repeated the analysis to consider a broader definition of neighborhood using destinations within 1-mile of the residence. To assess potential exposure misclassification due to relocation following stroke, we repeated the analysis to exclude participants who indicated they had moved at the time of the 3-month interview. Relocation information was only known for patients enrolled in 2014–2019, so the study population was restricted to these years (*N* = 1, 111) for this sub analysis.

## Results

**Fig. 1.**
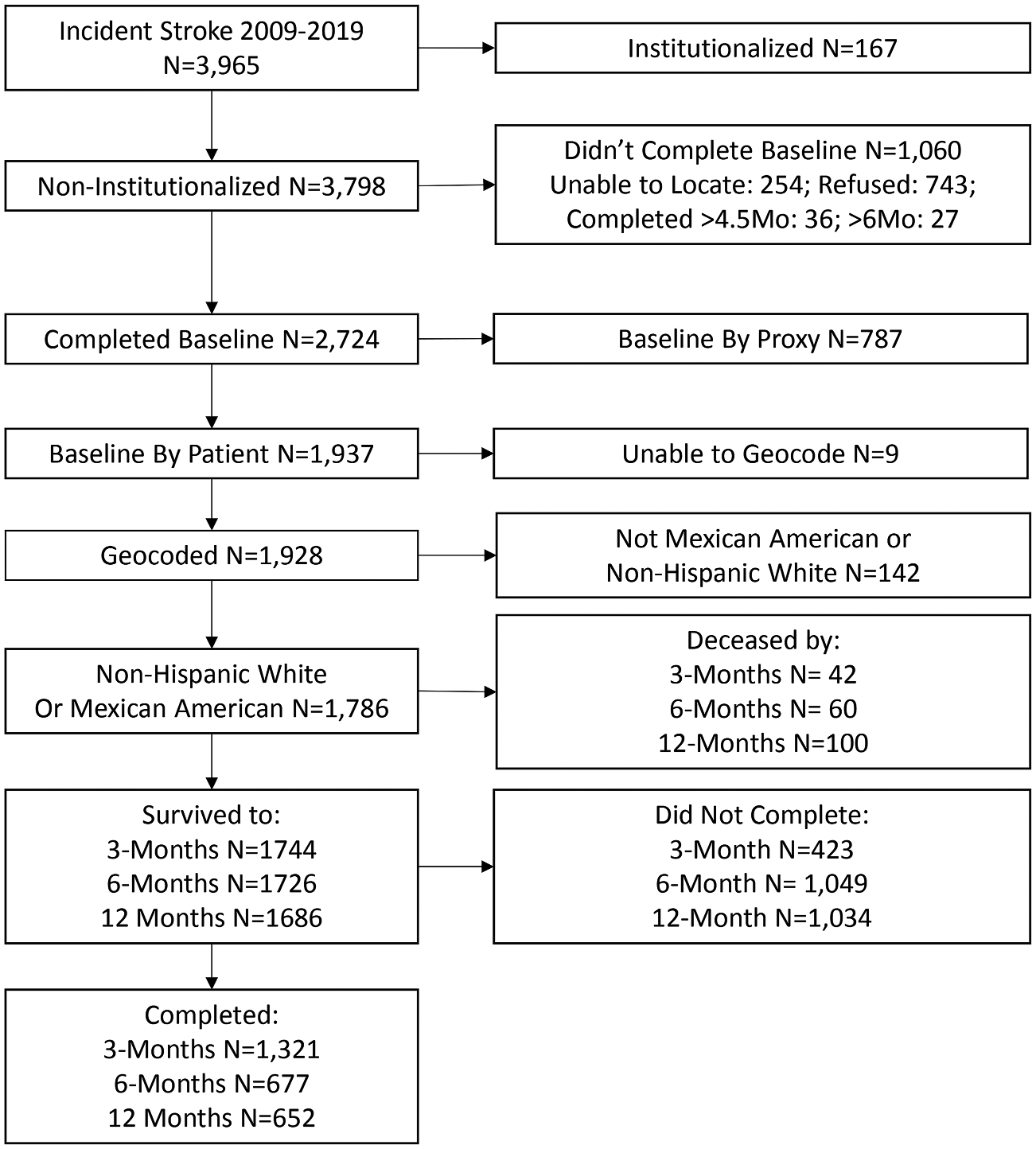
Eligibility flow chart. All numbers provided are those included for functioning outcome (2009–2019). Quality of life measure was obtained from 2010 to 2019 (Completed: 3-Month *N* = 1,222, 6-Month *N* = 677, 12-Month *N* = 652). Depression measure was obtained from 2011 to 2019 (Completed: 3-Month *N* = 1,086, 6-Month *N* = 662, 12-Month *N* = 631). Only survivors enrolled in 2014 or later were eligible to complete the 6- and 12- month follow-up.

Figure 1 illustrates the impact of the eligibility criteria on the study population. Of 3,965 BASIC project enrollees with a first-time stroke (2009–2019), we excluded those institutionalized prior to stroke (*N* = 167), without a baseline interview (*N* = 1,074), with a proxy baseline interview (*N* = 787), and without a geocoded address (*N* = 9). We further restricted analyses to Mexican American (*N* = 1,114) or Non-Hispanic White (*N* = 672) survivors (excluded survivors self-identifying as Non-Hispanic Black [*N* = 98], Non-Hispanic American Indian [*N* = 16], Hispanic Asian [*N* = 10], Non-Hispanic Asian [*N* = 9], and Hispanic Black [*N* = 9]). The final analytic sample included 1,786 participants who completed at least one follow-up interview (3-month: *N* = 1,321; 6-month: *N* = 677; 12-month: *N* = 652). Outcome-specific analytic-samples are detailed in Figure 1.

**Fig. 2.**
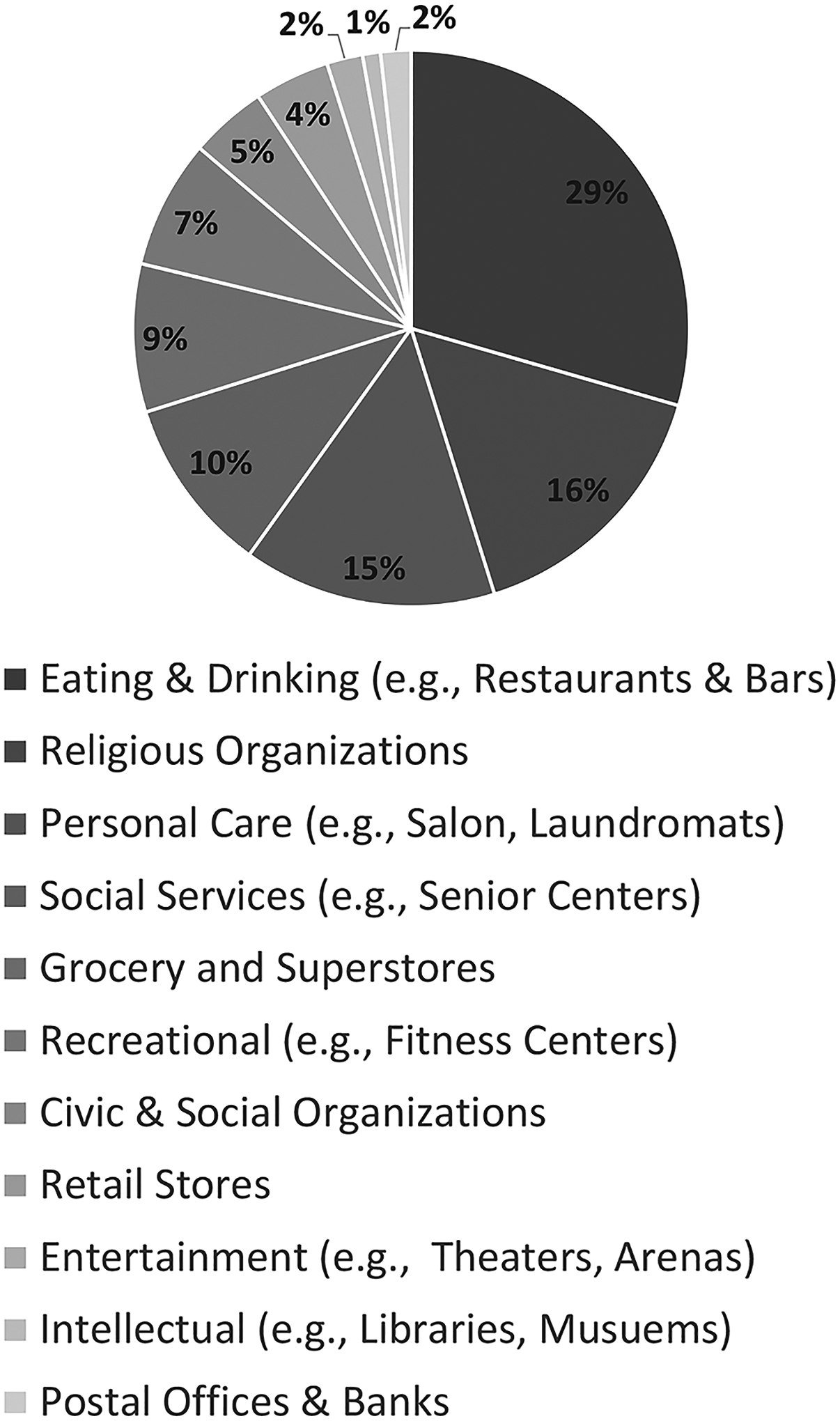
Destinations by type in the Corpus-Christi-Kingsville-Alice, TX combined statistical area, 2009–2019.

The median number of destinations within 0.5 miles of the home locations of our study participants was 29 (IQR=15, 44). Figure 2 portrays composition of the destinations in the Corpus Christi-Kingsville-Alice, TX combined statistical area, by type. During the study period (2009–2019), the most common destination types in this region were eating and drinking establishments (29.5%), religious organizations (15.7%), and personal care sites (14.7%). Our study population had a median age of 64.0 years (IQR=56.0, 73.0), was 55% male, 62.4% Mexican American, 50.5% married/cohabitating, 69.7% completed at least high school, 82.1% had insurance, 90.9% had experienced ischemic stroke, and 74% had mild stroke. A greater proportion of Mexican American (relative to non-Hispanic White), post-high school education, and uninsured survivors resided in neighborhoods with greater number of destinations (Table 1). Prior to their stroke, most had no to slight disability (86.5%, mRS≤2), no cognitive impairment (59.2%, IQCODE≤3), no depression (66.4%), median of 2 comorbidities (IQR=1, 3), did not use alcohol excessively (92.2%), and had never smoked (57.0%). A greater proportion of survivors had moderate-severe disability or cognitive impairment in neighborhoods with a greater number of destinations (Table 1). We also observed neighborhoods with greater counts of destinations had lower affluence, greater disadvantage, and greater ethnic/immigration based on the derived scores (Table 1). Median poststroke outcomes by quartile of destination count are presented in Table 2. Across the first-year poststroke, median ADL/IADL was 1.73 (IQR=1.82, 2.50), 3MSE was 90.00 (IQR=82.00, 95.00), SS-QoL was 3.75 (IQR=2.83, 4.50), and PHQ8 was 4.50 (IQR=1.00, 10.00). Poststroke ADL/IADL and SS-QoL differed by quartile of destination count for all timepoints; poststroke PHQ8 differed by quartile of destination count at 3-months (Table 2).

**Fig. 3.**
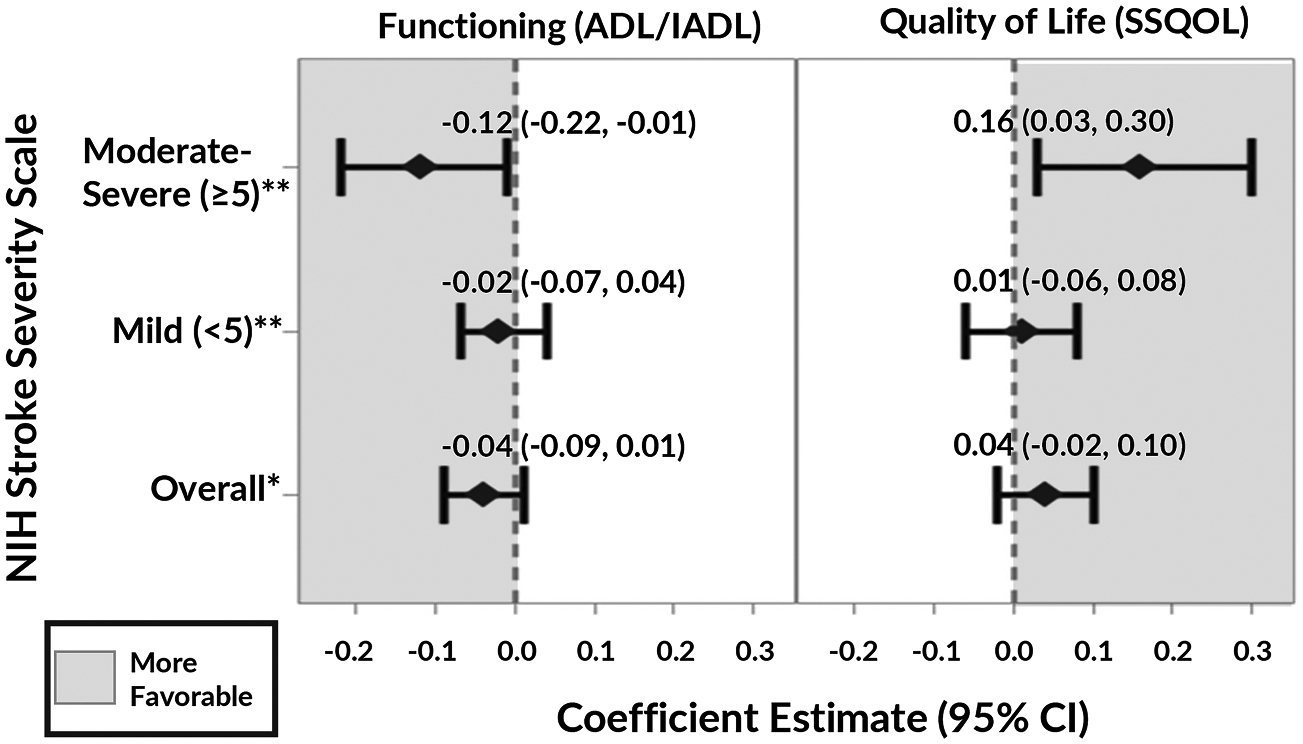
Association of destinations within 0.5 mile with poststroke functioning and quality of life in the 1st year. *Destination count was centered at median and standardized by interquartile range. Model is adjusted for follow-up timepoint, demographics (age, sex, race/ethnicity), individual socioeconomic status (education, health insurance), pre-stroke health (comorbidities, function, cognition, depression, smoking, excessive alcohol use), interpersonal factors (marital status, social support), neighborhood socioeconomic status (neighborhood disadvantage, neighborhood affluence), neighborhood ethnicity and immigration composition, and stroke factors (type of stroke, severity). **Results by stroke severity are additionally adjusted for interaction between destinations and stroke severity. ***ADL/IADL = Activities of Daily Living (ADL)/Instrumental ADL (range 1–4, lower is better), and SSQOL = Stroke-Specific Quality of Life scale (range, 0–5, higher is better).

Stroke severity appeared to modify the association of access to nearby destinations with poststroke functioning (Table 3, p-value for interaction term=0.0912) and quality of life (Table 4, p-value for interaction term=0.0475). Among those who experienced a mild stroke, greater number of destinations did not appear to be associated with post-stroke functioning or quality of life (Table 3 and 4). Among those who experienced a moderate-severe stroke, a greater number of destinations was associated with more favorable functioning after adjusting for confounders (mean difference in ADL/IADL= −0.1164, 95% CI= −0.2179, −0.0148, *p* = 0.0247; Fig. 3, Table 3, model 8) and quality of life (mean difference=0.1633, 95% CI=0.0255, 0.3011, *p* = 0.0202; Fig. 3, Table 4, model 8). We did not observe a three-way interaction between neighborhood destinations, stroke severity, and follow-up duration (*p* = 0.8949), or with neighborhood destinations and follow-up duration (*p* = 0.8311) with ADL/IADL. We also did not observe this three-way interaction (*p* = 0.2884), or an interaction with follow-up duration (*p* = 0.4498) for SS-QoL. Estimated differences and confidence intervals for a three-way interaction are presented in Tables 3 & 4 (Model 9) and demonstrated overlapping confidence intervals by follow-up time within each level of severity.

When evaluating the association of neighborhood destinations and cognition, there did not appear to be a 3-way or 2-way interaction with stroke severity and/or duration to follow-up (for 3-way interaction, *p* = 0.9084; for interaction with stroke severity, *p* = 0.5027; for interaction with follow-up duration, *p* = 0.3783). After adjusting for all confounders, the number of neighborhood destinations was not associated with poststroke cognition (mean difference=0.4845, 95% CI= −20.2117, 1.1807, *p* = 0.1723, Table 5). Similarly, when evaluating the association with poststroke depression, there appeared to be no 3-way or 2-way interactions with stroke severity and/or duration to follow-up (for 3-way interaction, *p* = 0.6320; for interaction with stroke severity, *p* = 0.8855; for interaction with follow-up duration, *p* = 0.9019). After adjusting for all confounders, the number of neighborhood destinations was not associated with poststroke depression (mean difference= −0.0262, 95% CI= −0.4428, 0.3904, *p* = 0.9019, Table 5).

The results of our sensitivity analysis are presented in Tables S1 and S2. When the neighborhood was expanded to 1 mile of the residence, we saw a similar association with ADL/IADL among survivors of a moderate-severe stroke (mean difference= −0.1475, 95% CI= −0.2639, −0.0312, *p* = 0.0130, Table S1). We did not see an association with SS-QoL among survivors of moderate-severe stroke survivors (mean difference=0.1030, 95% CI= −0.0560, 0.2620), although the confidence intervals overlap. In addition, we observed an association between neighborhood destinations defined by a 1-mile buffer and poststroke cognition among stroke survivors (mean difference=0.9471, 95% CI= −0.1213, 1.7730, *p* = 0.0247, Table S2). Again, we did not observe an association with post-stroke PHQ8. When restricting the analysis to those enrolled in 2014–2019 who had not indicated they moved (*N* = 1,066), we observed an association between count of neighborhood destinations and post-stroke ADL/IADL and SS-QoL among survivors of a moderate-severe stroke but not mild stroke (Table S1). Again, we did not observe an association with post-stroke 3MSE or PHQ8.

## Discussion

Among moderate-severe stroke survivors, residing within 0.5 miles of a greater number of destinations was associated with more favorable functioning and quality of life over the first year after stroke. These differences appear to persist across the first year, and the magnitude of the association was not modified by follow-up time; however, effect sizes were small (Hedges’ *g* < 0.20; Table S3).^37^ In our adjusted models, we were able to account for numerous mixed-level covariates, and it was only after adjusting for these likely confounders (including neighborhood-level SES) that we observed these associations. We did not observe that access to more neighborhood destinations to be associated with functioning or quality of life among mild stroke survivors. We also did not observe an association between destinations within a half mile and cognition or depression. When we considered the neighborhood to be defined as one mile within the residence, destinations continued to be favorably associated with functioning but not quality of life among moderate-severe stroke survivors. We also observed a favorable association with cognition among stroke survivors; however, the estimated mean difference in outcome was small.

This study contributes to limited research on neighborhood destinations and poststroke outcomes. A previous publication utilizing BASIC data from 2009 to 2019, which defined the neighborhood by residential census tract, identified associations with poststroke outcomes but only considered them at 3-months poststroke.^25^ Again, we confirmed stroke severity as an effect modifier of the impact of the neighborhood on poststroke functioning and quality of life.^23,25,30^ As discussed by Stulberg et al., there is a stronger expected association among those who survive more severe stroke, as their mobility is likely to be more constrained as compared to someone with a milder stroke.^10,11,30^ In this study, we observed the association between destinations with functioning and quality of life persists through the first year poststroke among moderate-severe stroke survivors. In a previous publication, we noted the implications of the potential benefit of the neighborhood environment on rehabilitation practices for stroke survivors residing in the community.^25^ Qualitative research suggests that there is limited consideration of the neighborhood environment in current rehabilitation efforts.^24,38^ Considering the potential enduring benefits of the neighborhood environment, researchers and clinicians might explore ways to integrate neighborhood destinations into rehabilitation efforts.

Our study has some important limitations: The decision to define the neighborhood using a 0.5-mile buffer was subjective and additional research is needed to explore the most appropriate distance to consider for stroke survivors. We did address this limitation in part by considering a 1-mile buffer, but it is possible a shorter distance may be more walkable and thus more appropriate. We also considered proximity to a variety of types of destinations, it is possible that access to certain types of destinations have varying effects on poststroke outcomes.^25^ Additionally, we did not assess the accessibility of the destinations (e.g., walkability), or whether residing near neighborhood destinations translated to survivors frequenting these destinations. Furthermore, we did not evaluate the specific mechanisms by which how neighborhood destinations may influence poststroke outcomes, such as physical activity or socialization.^14,15^ We also note the potential for confounding related to individual-level factors not controlled for due to the potential for these factors to be related to self-selection into the neighborhood.^39^ We similarly recognize potential confounding at the neighborhood level for factors related to perceived safety, street aesthetics, or accessibility of sidewalks or street crossings.^20,26,40–42^ Lastly, this study may not be generalizable to stroke survivors outside of Nueces County, Texas. It may not be generalizable to survivors residing in regions with more rural areas, different climates, or different cultural composition.

In conclusion, we find that access to greater number of destinations within 0.5 or 1 mile of the survivor’s residence was associated with more favorable functioning and within 0.5 miles with quality of life among survivors of a moderate-severe stroke and this association persisted through the first year poststroke. Future research is needed to confirm these findings in other study populations, to explore what proximity of destinations is relevant to the stroke survivor, consider the influence of specific types of destinations, and investigate potential mechanisms by which proximity to destinations supports poststroke outcomes.

Generative AI and AI-assisted technologies were NOT used in the preparation of this work.

## Supplementary Material

1

Supplementary material associated with this article can be found, in the online version, at doi:10.1016/j.jstrokecerebrovasdis.2026.108545.

## Figures and Tables

**Table 1:** Stroke survivor characteristics by density of destinations (*N* = 1,786).

Characteristic	1^st^ Quartile of Destinations (*N* = 441) Median (IQR) or n (%)	2^nd^ Quartile of Destinations (*N* = 455) Median (IQR) or n (%)	3^rd^ Quartile of Destinations (*N* = 438) Median (IQR) or n (%)	4^th^ Quartile of Destinations (*N* = 452) Median (IQR) or n (%)	P-Value
Destination Count with 0.5 Miles	8 (3, 11)	21 (18, 25)	36 (34, 40)	53 (48, 65)	<0.0001^1^
Age in years	65.0 (57.0, 74.0)	64.0 (56.0, 72.0)	64.0 (56.0, 74.0)	63.0 (55.5, 73.0)	0.2367^1^
Sex					
Male	253 (57.4%)	251 (55.2%)	243 (55.5%)	235 (52.0%)	
Female	188 (42.6%)	204 (44.8%)	195 (44.5%)	217 (48.0%)	0.4404^2^
Race/Ethnicity					
Mexican American	226 (51.3%)	278 (61.1%)	313 (71.5%)	297 (65.7%)	
Non-Hispanic White Education Attainment	215 (48.8%)	177 (38.9%)	125 (28.5%)	155 (34.3%)	<0.0001^2^
< High School	110 (24.9%)	134 (29.5%)	139 (31.7%)	158 (35.0%)	
High School	134 (30.4%)	124 (27.3%)	137 (31.3%)	140 (31.0%)	
> High School	197 (44.7%)	197 (43.3%)	162 (37.0%)	154 (34.1%)	0.0062^2^
Health Insurance Status^3^					
Insured	376 (86.4%)	364 (81.4%)	358 (82.9%)	347 (77.6%)	
No Insurance	59 (13.6%)	83 (18.6%)	74 (17.1%)	100 (22.4%)	0.0076^2^
Modified Rankin Scale Score^3^					
No disability	174 (39.8%)	160 (35.8%)	149 (34.6%)	139 (31.1%)	
No significant disability	72 (16.5%)	89 (19.9%)	63 (14.6%)	93 (20.8%)	
Slight disability	150 (34.3%)	131 (29.3%)	158 (36.7%)	146 (32.7%)	
Moderate disability	33 (7.6%)	37 (8.3%)	42 (9.7%)	42 (9.4%)	
Moderately severe to severe disability	8 (1.8%)	30 (6.7%)	19 (4.4%)	27 (6.0%)	0.0034^2^
Pre-Stroke IQCODE^3^	3.0 (3.0, 3.1)	3.0 (3.0, 3.1)	3.0 (3.0, 3.2)	3.0 (3.0, 3.3)	0.0234^1^
Pre-Stroke Depression^3^					
No Depression	313 (71.5%)	295 (65.1%)	278 (64.1%)	289 (64.9%)	
Depression diagnosis or Antidepressant Use	125 (28.5%)	158 (34.9%)	156 (35.9%)	156 (35.1%)	0.0768^2^
Comorbid Score	2.0 (1.0, 3.0)	3.0 (1.0, 4.0)	2.0 (1.0, 3.0)	2.0 (1.0, 3.0)	0.1617^1^
Excessive alcohol use					
Not Indicated	404 (91.6%)	424 (93.2%)	409 (93.4%)	410 (90.7%)	
Indicated	37 (8.4%)	31 (6.8%)	29 (6.6%)	42 (9.3%)	0.3793^2^
Smoking Status^3^					
Never smoked	258 (58.5%)	255 (56.2%)	259 (59.1%)	245 (54.3%)	
Ever smoker	183 (41.5%)	199 (43.8%)	179 (40.9%)	206 (45.7%)	0.4461^2^
Marital Status					
Married/ Cohabitating	249 (56.5%)	225 (49.5%)	223 (50.9%)	205 (45.4%)	
Divorced/ Separated	86 (19.5%)	110 (24.2%)	94 (21.5%)	117 (25.9%)	
Widowed	76 (17.2%)	82 (18.0%)	77 (17.6%)	80 (17.7%)	
Single/Never Married	30 (6.8%)	38 (8.4%)	44 (10.1%)	50 (11.1%)	0.0762^2^
Social Support Scale^3^	2.3 (1.9, 2.6)	2.3 (1.9, 2.6)	2.3 (1.9, 2.6)	2.3 (1.9, 2.6)	0.2914^1^
Neighborhood Affluence Score	0.30 (0.18, 0.36)	0.26 (0.13, 0.39)	0.22 (0.12, 0.30)	0.22 (0.12, 0.26)	<0.0001^1^
Neighborhood Disadvantage Score	0.12 (0.07, 0.16)	0.14 (0.12, 0.19)	0.15 (0.12, 0.19)	0.17 (0.12, 0.20)	<0.0001^1^
Ethnic Immigration Score	0.32 (0.25, 0.45)	0.36 (0.29, 0.48)	0.43 (0.34, 0.49)	0.38 (0.34, 0.49)	<0.0001^1^
Stroke Type					
Ischemic	403 (91.4%)	420 (92.3%)	391 (89.3%)	409 (90.5%)	
Intracerebral hemorrhage	38 (8.6%)	35 (7.7%)	47 (10.7%)	43 (9.5%)	0.4390^2^
Stroke Severity (NIHSS)					
Mild (<5)	345 (78.6%)	324 (71.7%)	319 (73.8%)	330 (73.2%)	
Moderate-Severe (≥5)	94 (21.4%)	128 (28.3%)	113 (26.2%)	121 (26.8%)	0.1024^2^

Abbreviations: IQR – Interquartile Range; IQCODE – Informant Questionnaire on Cognitive Decline in the Elderly; NIHSS – NIH Stroke Scale, FU – Follow-Up; ADL/IADL – Activities of Daily Living/Instrumental ADL; 3MSE – Modified Mini-Mental State Examination; SS-QoL – abbreviated Stroke Specific Quality of Life Scale; PHQ-8 – Patient Health Questionnaire Eight.

1 Analysis of Variance Test.

2 Chi-Squared Test.

3 Missing baseline data at for following variables: health insurance status (*n* = 25), modified Rankin score (*n* = 24), IQCODE (*n* = 355), pre-stroke depression (*n* = 16), smoking status (*n* = 2), social support score (*n* = 11), Stroke Severity (NIHSS) (*n* = 12).

**Table 2:** Outcomes among stroke survivors by density of destinations (*N* = 1,786).

Characteristic	1^st^ Quartile of Destinations (*N* = 441) Median (IQR) or n (%)	2^nd^ Quartile of Destinations (*N* = 455) Median (IQR) or n (%)	3^rd^ Quartile of Destinations (*N* = 438) Median (IQR) or n (%)	4^th^ Quartile of Destinations (*N* = 452) Median (IQR) or n (%)	P-Value
Survived to 3-Mo FU	427 (96.8%)	445 (97.8%)	430 (98.2%)	442 (97.8%)	0.5909^1^
Completed 3-Mo FU					
Not Completed	116 (27.2%)	86 (19.3%)	107 (24.9%)	114 (25.8%)	
Completed	311 (72.8%)	359 (80.7%)	323 (75.1%)	328 (74.2%)	0.0365^1,2^
3-Mo ADL/IADL^4^	1.59 (1.18, 2.36)	1.91 (1.21, 2.57)	1.93 (1.33, 2.64)	1.82 (1.23, 2.55)	0.0021^3^
3-Mo 3MSE^5^	91.0 (83.0, 95.0)	90.0 (83.0, 95.0)	88.0 (79.0, 94.0)	90.0 (81.0, 95.0)	0.0844^3^
3-Mo SS-QoL^6^	4.00 (3.00, 4.58)	3.67 (2.75, 4.42)	3.50 (2.67, 4.38)	3.75 (3.00, 4.46)	0.0028^3^
3-Mo PHQ-8^7^	4.00 (1.0, 9.0)	5.00 (2.0, 11.0)	6.0 (1.0, 13.0)	4.0 (1.0, 10.0)	0.0060^3^
Survived to 6-Mo FU	423 (95.9%)	443 (97.4%)	424 (96.8%)	436 (96.5%)	
Completed 6-Mo FU^1^					
Not Completed	96 (35.4%)	98 (36.2%)	93 (33.6%)	108 (42.7%)	
Completed	175 (64.6%)	173 (63.8%)	184 (66.4%)	145 (57.3%)	0.1528^1,2^
6-Mo ADL/IADL^4^	1.43 (1.09, 2.32)	1.82 (1.27, 2.55)	1.73 (1.14, 2.46)	1.59 (1.14, 2.41)	0.0852^3^
6-Mo 3MSE^5^	91.0 (94.0, 95.0)	90.0 (82.0, 94.0)	89.0 (78.0, 95.0)	90.0 (82.0, 95.0)	0.2413^3^
6-Mo SS-QoL^6^	4.08 (3.17, 4.67)	3.50 (2.67, 4.36)	3.58 (2.75, 4.50)	3.92 (2.92, 4.50)	0.0059^3^
6-Mo PHQ-8^7^	4.0 (1.0, 8.0)	5.0 (1.0, 9.0)	4.0 (1.0, 11.0)	5.0 (1.0, 10.0)	0.1605^3^
Survived to 12-Mo FU	414 (93.9%)	434 (95.4%)	411 (96.8%)	427 (94.5%)	0.7219^1^
Completed 12-Mo FU^1^					
Not Completed	108 (41.1%)	97 (36.7%)	100 (37.0%)	88 (35.5%)	
Completed	155 (58.94%)	167 (63.3%)	170 (63.0%)	160 (64.5%)	0.5854^1,2^
12-Mo ADL/IADL^4^	1.43 (1.09, 2.23)	1.96 (1.36, 2.55)	1.82 (1.23, 2.50)	1.64 (1.14, 2.48)	0.0196^3^
12-Mo 3MSE^5^	92.0 (85.0, 96.0)	90.0 (82.0, 95.0)	89.0 (81.0, 95.0)	90.0 (80.0, 96.0)	0.3515^3^
12-Mo SS-QoL^6^	4.17 (3.00, 4.75)	3.42 (2.75, 4.50)	3.67 (2.75, 4.50)	3.75 (2.92, 4.58)	0.0147^3^
12-Mo PHQ-8^7^	3.0 (1.0, 8.0)	5.0 (1.0, 10.5)	5.0 (1.0, 12.0)	4.0 (1.0, 10.0)	0.3312^3^

Abbreviations: IQR – Interquartile Range; IQCODE – Informant Questionnaire on Cognitive Decline in the Elderly; NIHSS – NIH Stroke Scale, FU – Follow-Up; ADL/IADL – Activities of Daily Living/Instrumental ADL; 3MSE – Modified Mini-Mental State Examination; SS-QoL – abbreviated Stroke Specific Quality of Life Scale; PHQ-8 – Patient Health Questionnaire Eight.

1 Chi-Squared Test.

2 Counts, percentages, and analysis only considers those eligible at and surviving to the indicated timepoint. Only survivors enrolled in 2014 or later were eligible to complete the 6- and 12- month follow-up.

3 Analysis of Variance Test.

4 Study Population for ADL/IADL included all enrolled 2009–2019 who completed the follow-up (3-Mo: *N* = 1321, 6-Mo: *N* = 677, 12-Mo: 652); the ADL/IADL was missing for following time-points 3-Mo (*n* = 21), 6-Mo (*n* = 5), 12-Mo (*n* = 6).

5 Study Population for 3MSE included all enrolled 2009–2019 who completed the follow-up, excluding those done by proxy (3-Mo: *N* = 1284, 6-Mo: *N* = 662, 12-Mo: 631); the 3MSE was missing for following time-points 3-Mo (*n* = 60), 6-Mo (*n* = 41), 12-Mo (*n* = 60).

6 Study Population for SS-QoL included all enrolled 2010–2019 who completed the follow-up (3-Mo: *N* = 1222, 6-Mo: *N* = 677, 12-Mo: 652); the SS-QoL was missing for following time-points 3-Mo (*n* = 50), 6-Mo (*n* = 1), 12-Mo (*n* = 2).

7 Study Population for PHQ-8 included all enrolled 2011–2019 who completed the follow-up, excluding those done by proxy (3-Mo: *N* = 1086, 6-Mo: *N* = 662, 12-Mo: 631); the PHQ-8 was missing for following time-points 3-Mo (*n* = 11), 6-Mo (*n* = 2), 12-Mo (*n* = 8).

**Table 3:** Difference in post-stroke ADL/IADL within first year associated with an interquartile range difference in counts of destinations within 0.5 miles of the stroke survivor's residence accounting for an interaction with stroke-severity^1^.

	Mild Stroke (NIHSS < 5)		Moderate-Severe Stroke (NIHSS ≥ 5)	
Model^2^	IQR Difference (95% CI)	P-Value	IQR Difference (95% CI)	P-Value
1: Stroke Severity	0.0449 (−0.0142, 0.1040)	0.1368	−0.0258 (−0.1376, 0.0861)	0.6517
2: 1 + Demographics	0.0251 (−0.0320, 0.0822)	0.3890	−0.0590 (−0.1663, 0.0483)	0.2808
3: 2 + Individual SES	0.0175 (−0.0395, 0.0744)	0.5476	−0.0630 (−0.1701, 0.0442)	0.2492
4: 3 + Pre-Stroke Health	0.0040 (−0.0498, 0.0578)	0.8835	−0.0901 (−0.1915, 0.0114)	0.0818
5: 4 + Pre-Stroke Behaviors	0.0040 (−0.0498, 0.0579)	0.8832	−0.0876 (−0.1894, 0.0140)	0.0911
6: 5 + Interpersonal Factors	0.0004 (−0.0535, 0.0544)	0.9874	−0.0844 (−0.1859, 0.0172)	0.1033
7: 6 + Neighborhood SES	−0.0177 (−0.0721, 0.0366)	0.5218	−0.1113 (−0.2129, −0.0097)	0.0319
8: 7 + Stroke Type	−0.0182 (−0.0725, 0.0360)	0.5100	−0.1164 (−0.2179, −0.0148)	0.0247
9: 8 + Follow-Up Time Interaction				
3-Month	−0.0185 (−0.0727, 0.0358)	0.5045	−0.1162 (−0.2179, −0.0146)	0.0250
6-Month	−0.0316 (−0.1203, 0.0571)	0.4846	−0.1176 (−0.2913, 0.0560)	0.1842
12-Month	−0.0098 (−0.0987, 0.0792)	0.8298	−0.0770 (−0.2535, 0.0995)	0.3923

Abbreviations: ADL/IADL – Activities of Daily Living/Instrumental ADL; IQR – Interquartile Range; SES – Socioeconomic Status.

1 IQR Difference was computed for each stratum of stroke severity by using coefficients for the counts of destinations and interaction term for stroke severity.

2 Unadjusted model sequentially adjusted for 1) Stroke severity and interaction between destination count and stroke severity, 2) age quartile, sex, race/ethnicity; 3) education attainment, insurance; 4) modified Rankin scale, informant questionnaire on cognitive decline in the elderly, depression, comorbidity score, 5) excessive alcohol use, smoking; 6) marital status, social support score; 7) neighborhood disadvantage score, neighborhood affluence score, neighborhood ethnic/immigrant score; 8) stroke type.

**Table 4:** Difference in post-stroke SS-QoL within first year associated with an interquartile range difference in counts of destinations within 0.5 miles of the stroke survivor's residence accounting for an interaction with stroke-severity^1^.

	Mild Stroke (NIHSS < 5)		Moderate-Severe Stroke (NIHSS ≥ 5)	
Model^2^	IQR Difference (95% CI)	P-Value	IQR Difference (95% CI)	P-Value
1: Stroke Severity	−0.0541 (−0.1298, 0.0217)	0.1616	0.0372 (−0.1152, 0.1897)	0.6314
2: 1 + Demographics	−0.0376 (−0.1120, 0.0367)	0.3212	0.0864 (−0.0636, 0.2364)	0.2583
3: 2 + Individual SES	−0.0226 (−0.0967, 0.0515)	0.5498	0.0948 (−0.0547, 0.2444)	0.2134
4: 3 + Pre-Stroke Health	−0.0124 (−0.0819, 0.0572)	0.7274	0.1358 (−0.0036, 0.2752)	0.0562
5: 4 + Pre-Stroke Behaviors	−0.0120 (−0.0815, 0.0575)	0.7353	0.1420 (0.0022, 0.2817)	0.0465
6: 5 + Interpersonal Factors	−0.0121 (−0.0813, 0.0571)	0.7322	0.1330 (−0.0059, 0.2718)	0.0605
7: 6 + Neighborhood SES	0.0075 (−0.0620, 0.0770)	0.8324	0.1578 (0.0200, 0.2955)	0.0248
8: 7 + Stroke Type	0.0078 (−0.0617, 0.0773)	0.8256	0.1633 (0.0255, 0.3011)	0.0202
9: 8 + Follow-Up Time Interaction				
3-Month	0.0085 (−0.0610, 0.0779)	0.8114	0.1633 (0.0258, 0.3008)	0.0200
6-Month	0.0380 (−0.0743, 0.1503)	0.5074	0.1965 (−0.0280, 0.4210)	0.0861
12-Month	−0.0464 (−0.1583, 0.0655)	0.4164	0.2571 (0.0295, 0.4847)	0.0269

Abbreviations: SS-QoL – abbreviated Stroke Specific Quality of Life Scale; IQR – Interquartile Range; SES – Socioeconomic Status.

1 IQR Difference was computed for each stratum of stroke severity by using coefficients for the counts of destinations and interaction term for stroke severity.

2 Unadjusted model sequentially adjusted for 1) Stroke severity and interaction between destination count and stroke severity, 2) age quartile, sex, race/ethnicity; 3) education attainment, insurance; 4) modified Rankin scale, informant questionnaire on cognitive decline in the elderly, depression, comorbidity score, 5) excessive alcohol use, smoking; 6) marital status, social support score; 7) neighborhood disadvantage score, neighborhood affluence score, neighborhood ethnic/immigrant score; 8) stroke type.

**Table 5:** Difference in post-stroke 3MSE and PHQ-8 within first year associated with an interquartile range difference in counts of destinations within 0.5 miles of the stroke survivor's residence.

	3MSE		PHQ-8	
Model^2^	IQR Difference (95% CI)	P-Value	IQR Difference (95% CI)	P-Value
1: Unadjusted	−0.3683 (−1.1637, 0.4271)	0.3639	0.3488 (−0.1152, 0.8128)	0.1406
2: 1 + Demographics	−0.1510 (−0.8973, 0.5953)	0.6915	0.2132 (−0.2369, 0.6634)	0.3532
3: 2 + Individual SES	0.0512 (−0.6554, 0.7479)	0.8853	0.1287 (−0.3180, 0.5753)	0.5723
4: 3 + Pre-Stroke Health	0.1353 (−0.5573, 0.8279)	0.7014	0.0363 (−0.3773, 0.4500)	0.8633
5: 4 + Pre-Stroke Behaviors	0.1623, −0.5299, 0.8545)	0.6454	0.0265 (−0.3869, 0.4398)	0.9002
6: 5 + Interpersonal Factors	0.2557 (−0.4338, 0.9451)	0.4669	0.0518 (−0.3620, 0.4655)	0.8063
7: 6 + Neighborhood SES	0.4324 (−0.2667, 1.1316)	0.2251	−0.0068 (−0.4244, 0.4108)	0.9745
8: 7 + Stroke Severity & Type	0.4845 (−0.2117, 1.1807)	0.1723	−0.0262 (−0.4428, 0.3904)	0.9019
9: 8 + Stroke Severity Interaction				
Mild^2^	0.3662 (−0.4024, 1.1347)	0.3501	−0.0113 (−0.4734, 0.4509)	0.9619
Moderate-Severe^2^	0.9258 (−0.5591, 2.4106)	0.2213	−0.0863 (−1.0083, 0.8356)	0.8544
10: 8 + Follow-Up Time Interaction				
3-Month^2^	0.5119 (−0.1864, 1.2101)	0.1505	−0.0341 (−0.4575, 0.3893)	0.8744
6-Month^2^	0.3760 (−0.8025, 1.5545)	0.5316	0.0830 (−0.6226, 0.7886)	0.8177
12-Month^2^	−0.2407 (−1.4469, 0.9656)	0.6956	−0.0340 (−0.7507, 0.6827)	0.9259

Abbreviations: 3MSE – Modified Mini-Mental State Examination; PHQ-8 – Patient Health Questionnaire Eight; IQR – Interquartile Range; SES – Socioeconomic Status;.

1 1) Unadjusted model sequentially adjusted for 2) age quartile, sex, race/ethnicity; 3) education attainment, insurance; 4) modified Rankin scale, informant questionnaire on cognitive decline in the elderly, depression, comorbidity score, 5) excessive alcohol use, smoking; 6) marital status, social support score; 7) neighborhood disadvantage score, neighborhood affluence score, neighborhood ethnic/immigrant score; 8) stroke severity and type. Interaction terms applied to model 8 for 9) stroke severity and 10) follow-up time.

2 IQR Difference was computed for each stratum of the interaction term by using coefficients for the counts of destinations and interaction term.

## References

[1] Imoisili OE, Chung A, Tong X, Hayes DK, Loustalot F. Prevalence of stroke - behavioral risk Factor Surveillance System, United States, 2011–2022. MMWR Morb Mortal Wkly Rep. 2024;73(20):449–455. 10.15585/mmwr.mm7320a1. 38781110 PMC11115433

[2] Martin SS, Aday AW, Almarzooq ZI, 2024 Heart Disease and stroke statistics: a report of US and global data from the American Heart Association. Circulation. 2024;149(8):e347–e913. 10.1161/CIR.0000000000001209. 38264914 PMC12146881

[3] Lisabeth LD, Sanchez BN, Baek J, Neurological, functional, and cognitive stroke outcomes in Mexican Americans. Stroke. 2014;45(4):1096–1101. 10.1161/STROKEAHA.113.003912. 24627112 PMC3966956

[4] Huang YY, Chen SD, Leng XY, Post-stroke cognitive impairment: epidemiology, risk factors, and management. J Alzheimers Dis. 2022;86(3):983–999. 10.3233/JAD-215644. 35147548

[5] Lisabeth LD, Brown DL, Dong L, Outcomes in the year after first-ever ischemic stroke in a Bi-ethnic population. Ann Neurol. 2023;93(2):348–356. 10.1002/ana.26513. 36134521 PMC9892337

[6] Guo J, Wang J, Sun W, Liu X. The advances of post-stroke depression: 2021 update. J Neurol. 2022;269(3):1236–1249. 10.1007/s00415-021-10597-4. 34052887

[7] Tsao CW, Aday AW, Almarzooq ZI, Heart disease and stroke statistics-2022 update: a report from the American Heart Association. Circulation. 2022;145(8):e153–e639. 10.1161/CIR.0000000000001052. 35078371

[8] Winstein CJ, Stein J, Arena R, Guidelines for adult stroke rehabilitation and recovery: a guideline for healthcare professionals from the american heart association/american stroke association. Stroke. 2016;47(6):e98–e169. 10.1161/STR.0000000000000098. 27145936

[9] Guo Y, Zhang Z, Lin B, The unmet needs of community-dwelling stroke survivors: a systematic review of Qualitative studies. Int J Environ Res Public Health. 2021;18(4). 10.3390/ijerph18042140. PMC7926407 33671734

[10] Nakao M, Izumi S, Yokoshima Y, Matsuba Y, Maeno Y. Prediction of life-space mobility in patients with stroke 2 months after discharge from rehabilitation: a retrospective cohort study. Disabil Rehabil. 2020;42(14):2035–2042. 10.1080/09638288.2018.1550533. 30676134

[11] Tsunoda S, Shimizu S, Suzuki Y, Longitudinal changes in life-space mobility and the factors influencing it among chronic community-dwelling post-stroke patients. Disabil Rehabil. 2021:1–5. 10.1080/09638288.2021.2001054. Published online. 34894964

[12] Chen T, Zhang B, Deng Y, Fan JC, Zhang L, Song F. Long-term unmet needs after stroke: systematic review of evidence from survey studies. BMJ Open. 2019;9(5), e028137. 10.1136/bmjopen-2018-028137. PMC6530326 31110106

[13] Lin BL, Mei YX, Wang WN, Unmet care needs of community-dwelling stroke survivors: a systematic review of quantitative studies. BMJ Open. 2021;11(4), e045560. 10.1136/bmjopen-2020-045560. PMC8061855 33879490

[14] Jellema S, van Hees S, Zajec J, van der Sande R, Nijhuis-van der Sanden MW, Steultjens EM. What environmental factors influence resumption of valued activities post stroke: a systematic review of qualitative and quantitative findings. Clin Rehabil. 2017;31(7):936–947. 10.1177/0269215516671013. 27681480 PMC5482381

[15] Espernberger KR, Fini NA, Peiris CL. Personal and social factors that influence physical activity levels in community-dwelling stroke survivors: a systematic review of qualitative literature. Clin Rehabil. 2021;35(7):1044–1055. 10.1177/0269215521993690. 33586479

[16] Finlay J, Esposito M, Li M, Neighborhood active aging infrastructure and cognitive function: a mixed-methods study of older Americans. Prev Med. 2021;150. 10.1016/j.ypmed.2021.106669. PMC8316307 34087319

[17] Wu YT, Brayne C, Liu Z, Neighbourhood environment and dementia in older people from high-, middle- and low-income countries: results from two population-based cohort studies. BMC Public Health. 2020;20(1):1330. 10.1186/s12889-020-09435-5. 32873275 PMC7465327

[18] Besser LM, Rodriguez DA, McDonald N, Neighborhood built environment and cognition in non-demented older adults: the Multi-Ethnic Study of Atherosclerosis. Soc Sci Med. 2018;200:27–35. 10.1016/j.socscimed.2018.01.007. 29355828 PMC5893410

[19] Padeiro M, de Sao Jose J, Amado C, Neighborhood attributes and well-being among older adults in urban areas: a mixed-methods systematic review. Res Aging. 2021, 164027521999980. 10.1177/0164027521999980. Published online. PMC9039320 33906556

[20] Barnett A, Zhang CJP, Johnston JM, Cerin E. Relationships between the neighborhood environment and depression in older adults: a systematic review and meta-analysis. Int Psychogeriatr. 2018;30(8):1153–1176. 10.1017/S104161021700271X. 29223174

[21] Park YS, McMorris BJ, Pruinelli L, Song Y, Kaas MJ, Wyman JF. Use of geographic information systems to explore associations between neighborhood attributes and mental health outcomes in adults: a systematic review. Int J Environ Res Public Health. 2021;18(16). 10.3390/ijerph18168597. PMC8393279 34444345

[22] Townsend BG, Chen JT, Wuthrich VM. Barriers and facilitators to social participation in older adults: a systematic literature review. Clin Gerontol. 2021;44(4):359–380. 10.1080/07317115.2020.1863890. 33393443

[23] Delhey LM, Shi X, Morgenstern LB, Association of neighborhood recreation centers and poststroke outcomes in a population-based cohort. Stroke. 2023;54(10):2583–2592. 10.1161/STROKEAHA.122.041852. 37706339 PMC10530069

[24] Kylen M, Ytterberg C, von Koch L, Elf M. How is the environment integrated into post-stroke rehabilitation? A qualitative study among community-dwelling persons with stroke who receive home rehabilitation in Sweden. Health Soc Care Community. 2022;30(5):1933–1943. 10.1111/hsc.13572. 34541725

[25] Delhey LM, Shi X, Morgenstern LB, Neighborhood resources and health outcomes among stroke survivors in a population-based cohort. J Am Heart Assoc. 2024;13(14), e034308. 10.1161/JAHA.124.034308. 38958125 PMC11292760

[26] Twardzik E, Clarke PJ, Lisabeth LD, Enhanced street crossing features are associated with higher post-stroke physical quality of life. Top Stroke Rehabil. 2023;30(6):578–588. 10.1080/10749357.2022.2108970. 35924680 PMC9898471

[27] US Census-Bureau. Quick facts: nueces County, Texas. United States Census Bureau Quick facts. September 14, 2021. Accessed November 29, 2021. https://www.census.gov/quickfacts/nuecescountytexas.

[28] Smith MA, Risser JM, Moye LA, Designing multi-ethnic stroke studies: the brain attack surveillance in Corpus Christi (BASIC) project. Ethn Dis. 2004;14(4):520–526. https://www.ncbi.nlm.nih.gov/pubmed/15724771. 15724771

[29] von Elm E, Altman DG, Egger M, The strengthening the reporting of observational studies in epidemiology (STROBE) statement: guidelines for reporting observational studies. Epidemiology. 2007;18(6):800–804. 10.1097/EDE.0b013e3181577654. 18049194

[30] Stulberg EL, Twardzik E, Kim S, Association of neighborhood socioeconomic status with outcomes in patients surviving stroke. Neurology. 2021;96(21):e2599–e2610. 10.1212/WNL.0000000000011988. 33910941 PMC8205453

[31] Schlegel D, Kolb SJ, Luciano JM, Utility of the NIH stroke scale as a predictor of hospital disposition. Stroke. 2003;34(1):134–137. 10.1161/01.str.0000048217.44714.02. 12511764

[32] Melendez R, Clarke P, Khan A, Gomez-Lopez I, Li M, Chenoweth M. National neighborhood data archive (NaNDA): Socioeconomic status and demographic characteristics of census tracts. 2020. 10.3886/E119451V2, 2008–2017. Published online.

[33] Kroenke K, Strine TW, Spitzer RL, Williams JB, Berry JT, Mokdad AH. The PHQ-8 as a measure of current depression in the general population. J Affect Disord. 2009;114 (1–3):163–173. 10.1016/j.jad.2008.06.026. 18752852

[34] Post MW, Boosman H, van Zandvoort MM, Passier PE, Rinkel GJ, Visser-Meily JM. Development and validation of a short version of the stroke specific quality of life scale. J Neurol Neurosurg Psychiatry. 2011;82(3):283–286. 10.1136/jnnp.2009.196394. 20802211

[35] Rubin DB. Multiple Imputation for Nonresponse in Surveys. 99th ed. John Wiley & Sons; 2009.

[36] Thiese MS, Ronna B, Ott U. P value interpretations and considerations. J Thorac Dis. 2016;8(9):E928–E931. 10.21037/jtd.2016.08.16. 27747028 PMC5059270

[37] Durlak JA. How to select, calculate, and interpret effect sizes. J Pediatr Psychol. 2009;34(9):917–928. 10.1093/jpepsy/jsp004. 19223279

[38] Elf M, Rasoal D, Zingmark M, Kyĺen M. The importance of context-a qualitative study exploring healthcare practitioners’ experiences of working with patients at home after a stroke. BMC Health Serv Res. 2023;23(1):733. 10.1186/s12913-023-09735-7. 37415156 PMC10324136

[39] Lamb KE, Thornton LE, King TL, Methods for accounting for neighbourhood self-selection in physical activity and dietary behaviour research: a systematic review. Int J Behav Nutr Phys Act. 2020;17(1):45. 10.1186/s12966-020-00947-2. 32238147 PMC7115077

[40] Brenner AB, Burke JF, Skolarus LE. Moving toward an understanding of disability in older U.S. stroke survivors. J Aging Health. 2018;30(1):75–104. 10.1177/0898264316666125. 27605555 PMC5534387

[41] Ruiz LD, Brown M, Li Y, Neighborhood socioeconomic resources and crime-related psychosocial hazards, stroke risk, and cognition in older adults. Int J Environ Res Public Health. 2021;18(10). 10.3390/ijerph18105122. PMC8151671 34066049

[42] Twardzik E, Clarke P, Judd S, Colabianchi N. Neighborhood participation is less likely among older adults with sidewalk problems. J Aging Health. 2021;33(1–2):101–113. 10.1177/0898264320960966. 32960717 PMC8006802

