# Supplementary material for: Access to neighborhood destinations that offer opportunities for physical activity and socialization is associated with favorable post-stroke outcomes
Source: J Stroke Cerebrovasc Dis. Author manuscript; Available in PMC 2026 Jul 6. (PMC13334518; doi:10.1016/j.jstrokecerebrovasdis.2026.108545)
Supplement: 1 [file NIHMS2189683-supplement-1.pdf]

Table S1. Sensitivity Analysis to estimate IQR difference in post-stroke ADL/IADL, SS-QOL, 3MSE, and PHQ-8 within first year associated with an interquartile range difference in counts of destinations within 1 mile of the stroke survivor's residence (N=1,786).

**Model**	**IQR Difference (95% CI)**	**P-Value**
ADL/IADL: All Confounders + Stroke Severity Interaction		
Mild[2]	-0.0214 (-0.0844, 0.0416)	0.5054
Moderate-Severe[2]	-0.1475 (-0.2639, -0.0312)	0.0130
SS-QoL: All Confounders + Stroke Severity Interaction		
Mild[2]	0.0322 (-0.0498, 0.1142)	0.4415
Moderate-Severe[2]	0.1030 (-0.0560, 0.2620)	0.2036
3MSE: All Confounders	0.9471 (0.1213, 1.7730)	0.0247
PHQ8: All Confounders	-0.0408 (-0.5235, 0.4418)	0.8684

Abbreviations: 3MSE – Modified Mini-Mental State Examination; PHQ-8 – Patient Health Questionnaire Eight; IQR – Interquartile Range

[1] Adjusted for age quartile, sex, race/ethnicity, education attainment, insurance, modified Rankin scale, informant questionnaire on cognitive decline in the elderly, depression, comorbidity score, excessive alcohol use, smoking, marital status, social support score, neighborhood disadvantage score, neighborhood affluence score, neighborhood ethnic/immigrant score, stroke severity, stroke type, and interaction between stroke severity and neighborhood destinations.

[2] IQR Difference was computed for each stratum of the interaction term by using coefficients for the counts of destinations and interaction term.

Table S2. Sensitivity Analysis to estimate IQR difference in post-stroke ADL/IADL, SS-QOL, 3MSE, and PHQ-8 restricting the analysis to those who indicated they had not moved at the 3-month follow-up assessment (2014-2019, N=1,066).

**Final Model**	**IQR Difference (95% CI)**	**P-Value**
ADL/IADL: All Confounders[1] + Stroke Severity Interaction		
Mild[2]	0.0056 (-0.0578, 0.0690)	0.8624
Moderate-Severe[2]	-0.1508 (-0.2786, -0.0231)	0.0207
SS-QoL: All Confounders[1] + Stroke Severity Interaction		
Mild[2]	-0.0051 (-0.0873, 0.0771)	0.9033
Moderate-Severe[2]	0.1743 (0.0062, 0.3425)	0.0422
3MSE: All Confounders[1]	0.2387 (-0.5851, 1.0625)	0.5701
PHQ8: All Confounders[1]	0.1091 (-0.3694, 0.5875)	0.6550

Abbreviations: 3MSE – Modified Mini-Mental State Examination; PHQ-8 – Patient Health Questionnaire Eight; IQR – Interquartile Range

[1] Adjusted for age quartile, sex, race/ethnicity, education attainment, insurance, modified Rankin scale, informant questionnaire on cognitive decline in the elderly, depression, comorbidity score, excessive alcohol use, smoking, marital status, social support score, neighborhood disadvantage score, neighborhood affluence score, neighborhood ethnic/immigrant score, stroke severity, stroke type, and interaction between stroke severity and neighborhood destinations.

[2] IQR Difference was computed for each stratum of the interaction term by using coefficients for the counts of destinations and interaction term.

Table S3. Hedges' *g* for outcomes comparing first with fourth quartile of neighborhood resource density (across the first year)

	**Neighborhood Resource Density – First Quartile**		**Neighborhood Resource Density – Fourth Quartile**		
**Outcome**	**N[1]**	**Mean (SD)**	**N[1]**	**Mean (SD)**	**Hedges' *g***
ADL/IADL score	632	1.78 (0.77)	621	1.92 (0.83)	0.17
3MSE score	600	87.03 (12.85)	578	86.24 (12.29)	0.06
SS-QoL score	603	3.81 (0.95)	589	3.67 (0.97)	0.15
PHQ-8 score	581	5.62 (6.02)	564	6.46 (6.42)	0.14

Abbreviations: 3MSE – Modified Mini-Mental State Examination; PHQ-8 – Patient Health Questionnaire Eight; IQR – Interquartile Range

[1] N reflects the total number of follow-up assessments completed for the indicated measure across eligible participants.
